# Complex Monte Carlo Light-Driven Dynamics of Monomers in Functionalized Bond Fluctuation Model Polymer Chains

**DOI:** 10.3390/ma16124373

**Published:** 2023-06-14

**Authors:** Grzegorz Pawlik, Antoni C. Mitus

**Affiliations:** 1 Institute of Theoretical Physics, Wroclaw University of Science and Technology, 50-370 Wroclaw, Poland; antoni.mitus@pwr.edu.pl; 2 Social and Technical Sciences Faculty, Jan Wyzykowski University, 59-101 Polkowice, Poland

**Keywords:** bond-fluctuation model, photoinduced dynamics, azo-polymers, subdiffusion, superdiffusion

## Abstract

We study Monte Carlo dynamics of the monomers and center of mass of a model polymer chain functionalized with azobenzene molecules in the presence of an inhomogeneous linearly polarized laser light. The simulations use a generalized Bond Fluctuation Model. The mean squared displacements of the monomers and the center of mass are analyzed in a period of Monte Carlo time typical for a build-up of Surface Relief Grating. Approximate scaling laws for mean squared displacements are found and interpreted in terms of sub- and superdiffusive dynamics for the monomers and center of mass. A counterintuitive effect is observed, where the monomers perform subdiffusive motion but the resulting motion of the center of mass is superdiffusive. This result disparages theoretical approaches based on an assumption that the dynamics of single monomers in a chain can be characterized in terms of independent identically distributed random variables.

## 1. Introduction

Thin films of polymers functionalized with azobenzene molecules [1] exposed to an inhomogeneous linearly polarized laser light may develop a surface corrugation due to the light-driven motion of polymer chains. In Degenerate Two Wave Mixing experiments, a periodic pattern—Surface Relief Grating (SRG)—builds up [2,3]. The directed transport of functionalized chains, which takes place in the presence of an inhomogeneous light illumination, is attributed to a cooperative motion of the monomers, which results from multiple light-driven photoisomerization cycles trans↔cis of azobenzene molecules. Nevertheless, the detailed “microscopic” mechanisms responsible for laser light-driven mass transport in functionalized azo-polymers are poorly understood. This topic, which constitutes one of the challenges in the physics of polymers, was addressed by a number of theoretical and numerical models; see review papers [4,5,6]. The long sought-after model of the light-induced motion of azo-polymers, which directly accounts for the polymer matrix and reproduces the main effects of the build-up of SRG, including the fine structure of the density profile, was formulated by our group [7]. It is based on the Bond Fluctuation Model (BFM) (Section 2.1), generalized onto the case of polymer chains functionalized with azo-dyes (Section 2.2). The model offers a platform for modeling a wide class of nonlinear optics phenomena in host–guest systems, such as the inscription of various types of diffraction gratings, poling effects, or photoinduced dynamics. Those phenomena are characterized in terms of experimentally measurable quantities: diffraction efficiency, nonlinear susceptibilities, density profiles, or load parameters. The interested reader can find a detailed discussion of those topics, as well as the theoretical justification of the a priori assumptions made for BFM modeling of photoinduced motion of the azo-polymer chains, in a review paper [6].

Those studies open a promising perspective for the mathematical modeling of light-driven mass transport in azo-polymers. The long-term goal is an analytical model for a description of the light-induced dynamics of azo-chains. The first steps in this direction were presented in Ref. [8], where a physically naive (but mathematically advanced) modeling of the dynamics of the chains using the concept of Continuous Time Random Walk for independent random walkers in one dimension, reproduced the main features of SRG inscription, in particular its fine structure. The next steps of the modeling require, as an input, a more realistic and detailed characterization of the dynamics of single monomers as well as of the center of mass (CM) of a chain. One part of the problem was already solved [8]. Namely, a detailed study of the dynamics of CM of single chains has emphasized the importance of non-standard diffusion in light-driven mass transport. It displays not only various subdiffusive regimes, well-known from theoretical approaches [9,10] and computer simulations of standard (i.e., without guest molecules) BFM and other coarse-grained models [11,12,13,14,15,16,17,18,19,20,21], but also a superdiffusive regime, rare in physical systems. The complex dynamics of light-driven mass transport in azo-polymers is richer, and thus more challenging for a theoretical description of the underlying processes, than its counterpart for standard BFM chains. Those studies have formed the beginnings of an elegant hypothetical physical picture of the light-driven inscription of SRG, which assumes a dynamical coexistence of normal and anomalous diffusion (sub- and super-) in various parts of the system. This effect itself is an interesting contribution to the field of complexity in physical systems.

Unlike the case of the center of mass, the characterization of the complex light-driven dynamics of single monomers in the azo-polymer chain is still missing. The objective of the paper is to fill, at least partially, this gap. Methodologically, the paper is a continuation of a series of papers that use generalized BFM for functionalized polymer chains [6].

## 2. Materials and Methods

### 2.1. Monte Carlo Bond Fluctuation Model

In what follows, we briefly summarize the Bond Fluctuation Model and its Monte Carlo simulation; more details can be found in the review paper [6]. BFM, an important statistical physics non-specific lattice model for coarse-grained polymer chains, successfully reproduces a large variety of static and dynamic effects in dense polymer systems; see Refs. [22,23,24] for details. Each effective monomer/segment occupies a single lattice site. The nearest neighbor monomers are connected by links, which correspond closely to the Kuhn segments formed from the groups of monomers along the polymer chain [25]. In the three-dimensional (3D) model, five bond orientations with bond lengths (in lattice constants) are present [26]—2, $\sqrt{5}$, $\sqrt{6}$, 3, $\sqrt{10}$—with bond energies $E_{i}\;\left(i=1,\ldots ,5\right)$, $E_{i}=E_{0}\;\varepsilon_{i}$, where parameter $E_{0}$ sets an energy scale and defines the reduced temperature $T^{*}=k_{B}T/E_{0}$, and $\varepsilon_{i}$ denote dimensionless energies. In what follows, the reduced temperature will be denoted by *T* instead of $T^{*}$. The glass transition temperature for the model, estimated using geometrical as well as physical parameters, lies in the interval $0.225<T_{g}<0.3$ [27]. In this study, we investigate single polymer chains with N=25 monomers at temperature T=0.2, close to but below $T_{g}$.

**Figure 1:**
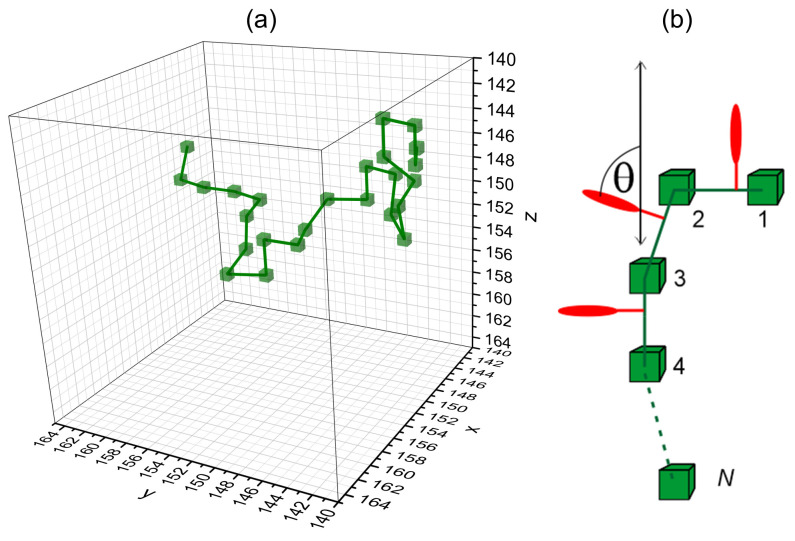
Exemplary configuration of a polymer chain consisting of N=25 monomers (green cubes) in 3D BFM model (**a**). Azo-dye molecules (red, not shown in (**a**)) are attached to each bond (**b**) (see text for more details).

In a single Monte Carlo step (MCS), each of the monomers performs a trial move of unit length along one of directions x,y,z. It is accepted that when (i), a new length of the bond does not violate imposed restrictions, (ii) the trial position of the monomer is neither occupied by a monomer nor by a nearest neighbor of a monomer, and (iii), the Metropolis acceptance rule does not reject the movement. In Figure 1, the exemplary conformation of the chain is presented [6].

### 2.2. Bond Fluctuation Model for Light–Matter Interaction for Functionalized Chains

In order to study the light-driven mass transport of polymer chains with attached azo-dye molecules, the BFM model was generalized to account for light–matter interaction [6,7,8]. The azo-dyes interacting with linearly polarized laser light undergo photoisomerization reactions trans↔cis with transition rate *p* [1]

(1) $$p=I_{L}\alpha \cos^{2}\theta ,$$

where $I_{L}$ and α denote, respectively, light intensity and the probability of a photoisomerization due to photon absorption. θ denotes an angle between the long axis (transition moment) of an azo-molecule in trans state and the direction of light polarization. In what follows, we use the reduced (dimensionless) light intensity $I=\alpha I_{L}$ [8]. The impact of Newtonian forces and torques, which result from photoisomerization reactions, on the chain, is mimicked in the model by an additional non-thermal trial movement (of unit length along one of the x,y,z axes) of the monomer closest to the dye. It is accepted with probability *p* (per MC step), Equation (1), if, additionally, (i) a new length of the bond does not violate imposed restrictions, (ii) the trial position of the monomer is neither occupied by a monomer nor by a nearest neighbor of a monomer. The Metropolis acceptance rule is not taken into account [8]. The geometry of the model is as follows; see Figure 1b. The dyes in trans state are assumed to be strictly perpendicular to the bond. Linearly polarized (along *z*-axis) light propagates in the *y*-direction; its intensity varies along the *x*-direction: I=I(x). Two simplifications in the modeling of photoisomerization cycles, originally introduced in Refs. [7,8], are used. Firstly, we neglect the effect of cis→trans reactions. Secondly, the $\cos^{2}\theta$ term in Equation (1) is replaced by a step function with value 0 for a small interval Δθ≪1 of angles around θ=π/2 and with value 1 for the remaining angles. The transition rate p(x) reads

(2) $$p\left(x\right)=\left\{\begin{array}{cc} I(x), & \theta \notin \Delta \theta ,\\ 0, & \theta \in \Delta \theta . \end{array}\right.$$

Transition rate p(x)=0 deactivates the photoisomerization transitions for the dyes which are nearly perpendicular to the bond.

Two light illumination setups I(x) are used. Constant illumination generates isotropic diffusion in space:

(3) $$I\left(x\right)=I_{0}.$$

Non-isotropic diffusion is studied using linear (along *x* axis) profile [8]:

(4) $$I\left(x\right)=I_{0}-\nabla I\;\left(x-x_{0}\right),$$

where $x_{0}$ denotes the center of lattice in the *x* direction and $I_{0}=I\left(x_{0}\right)$ denotes the intensity offset. The coefficient $\nabla I\equiv \frac{\partial I}{\partial x}$ is referred to as gradient; in this study it has a constant value $\frac{\partial I}{\partial x}=5\times 10^{-3}$.

To summarize, in a single MC step, each monomer performs two trial movements: a thermally-driven (Section 2.1) and a non-thermal one, characterized by the local intensity of light I(x).

### 2.3. Characterization of the Displacement of the Chain

The random walks performed by the monomers and the center of mass of a chain are characterized by their squared displacements from initial positions. The displacements are realizations of stochastic processes and, as such, display fluctuations. Thus, one introduces squared displacements averaged over an ensemble of $N_{0}$ independent realizations of random walks of single chains. In this paper, we study $N_{0}=10^{3}$ independent chains. Let vectors ${\vec{r}}_{i}^{\left(CM\right)}\left(t\right)$ and ${\vec{r}}_{i}^{\left(m\right)}\left(t\right)$ denote the positions of center of mass and monomer *m*, respectively, in *i*-th chain at time *t*. Then, the ensemble-averaged squared displacement reads

(5) $$<{\left(\Delta \vec{r}\right)}^{2}>\left(t\right)=\frac{1}{N_{0}}\sum_{i=1}^{N_{0}}{\left(\Delta {\vec{r}}_{i}\right)}^{2}\left(t\right),$$

where $\Delta {\vec{r}}_{i}\left(t\right)={\vec{r}}_{i}\left(t\right)-{\vec{r}}_{i}\left(0\right)$ and, for simplicity, we have omitted upper indices (CM) and (m).

**Figure 2:**
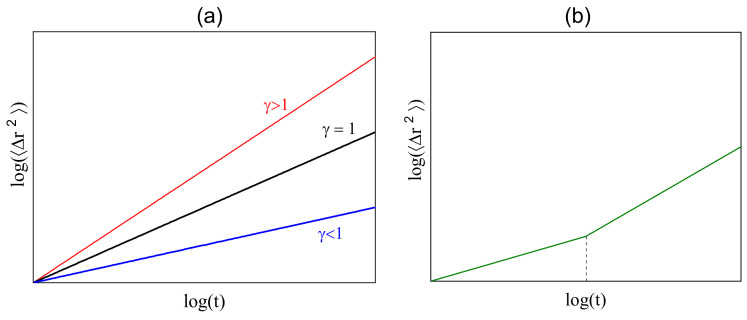
Power laws and complex dynamics. Plots of $<{\left(\Delta \vec{r}\right)}^{2}>\left(t\right)\propto t^{\gamma }$ in double logarithmic (**a**) scales. Red, black, and blue curves correspond to superdiffusion (γ>1), normal diffusion (γ=1), and subdiffusion (γ<1), respectively. Two scaling laws (**b**). The dashed line denotes the crossover between two regimes of anomalous diffusion, characterized by different values of exponent γ.

In the case when the dependence of $<{\left(\Delta \vec{r}\right)}^{2}>$ on time displays, in some interval of time, a power law with exponent γ

(6) $$<{\left(\Delta \vec{r}\right)}^{2}>\left(t\right)\propto t^{\gamma },$$

the dynamics of monomers/center of mass become complex. The value 0<γ<1 characterizes subdiffusion, γ>0 superdiffusion, and γ=1 represents a standard diffusion. Exponent γ is the slope of the double-logarithmic plot of $<{\left(\Delta \vec{r}\right)}^{2}>\left(t\right)$; see Figure 2a. In this way, we introduce the exponents $\gamma_{CM}$ (for the center of mass) and $\gamma_{m}$ (for *m*-th monomer) in the case of an isotropic in space dynamics:

(7) $$<{\left(\Delta {\vec{r}}^{\left(CM\right)}\right)}^{2}>\left(t\right)\propto t^{\gamma_{CM}},\;<{\left(\Delta {\vec{r}}^{\left(m\right)}\right)}^{2}>\left(t\right)\propto t^{\gamma_{m}}.$$

In the case of non-isotropic diffusion, the exponents along axes x,y,z can be different and the dynamics are characterized in terms of the “anisotropic” exponents $\gamma_{CM,x},\gamma_{CM,y}$ and $\gamma_{CM,z}$ corresponding to the diffusion of center of mass, and exponents $\gamma_{m,x},\gamma_{m,y}$ and $\gamma_{m,z}$-to the diffusion of *m*-th monomer. In this case, the displacements are calculated separately for each axis. For example,

(8) $$<{\left(\Delta x^{\left(CM\right)}\right)}^{2}>\left(t\right)\propto t^{\gamma_{CM,x}}.$$

In what follows, special attention will be paid to the dynamics of three objects: center of mass (CM), edge monomer (m=1), and central monomer (m=13).

The procedure for an estimation of the exponents γ in power laws requires some explanation. First, theoretical studies (see, e.g., [9,10]) predict a few anomalous diffusion regimes in various intervals of time. In the log–log plot of mean-square displacement a few linear parts are present with different values of γ, estimated from the slope in each interval of time (see Figure 2b). This method is used in an analysis of complex dynamics in computer simulations of polymer chains [11,12,13,15,16,17,28,29,30,31]. We follow the same route and estimate the values of exponent γ from the linear fits to log–log plots of <${\left(\Delta \vec{r}\right)}^{2}>\left(t\right)$. We have used this method in our earlier simulations of anomalous dynamics of polymer chains [8,32]. However, the interpretation of the results of the fitting procedure in terms of scaling laws becomes a delicate topic because the thumb rule requires that the power laws, such as Equation (6), should be valid at least in three decades. The time intervals of interest, reported in above mentioned papers, are shorter and constitute around 1.5 decades. Moreover, the approximation of the plots with straight lines may cause difficulties. In spite of those shortcomings, the authors interpret their results in terms of power laws and anomalous diffusion and verify theoretical predictions. We are of the opinion that the rigorous interpretation of the dynamics of polymer chains in terms of power laws should be treated with some care.

Although the analysis of the mechanisms responsible for various dynamical regimes constitutes a challenge in polymer physics, we restrict ourselves to an interval in which physical processes related to the mass transport in the polymer matrix take place. Motivated by the fact that the characteristic MC time interval for SRG formation constitutes approximately $t_{SRG}=5\times 10^{4}$ MCS [7], we chose the interval $(3\times 10^{3}$–$5\times 10^{4})$ MCS for an analysis.

## 3. Results

### 3.1. Homogeneous Illumination: $I\left(x\right)=I_{0}$

#### 3.1.1. Trajectories

**Figure 3:**
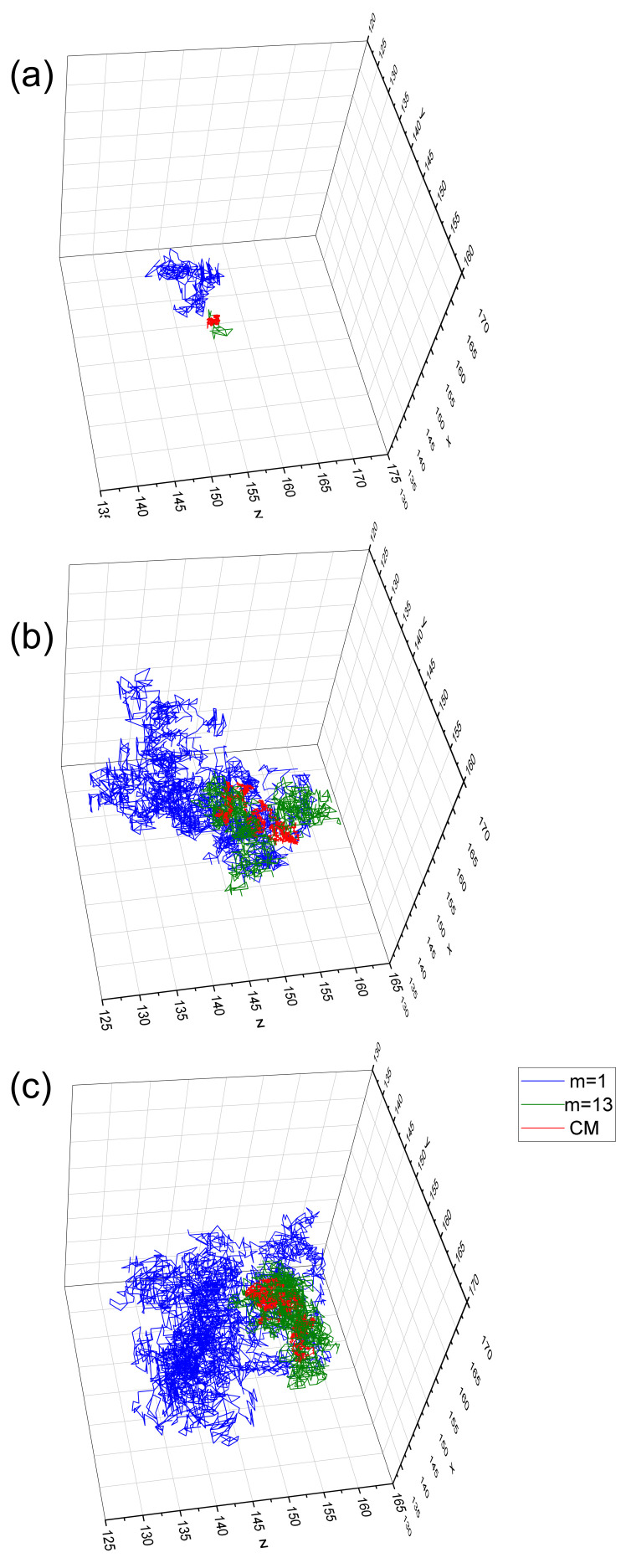
Exemplary trajectories of selected monomers and the CM of the chain in the full simulation period $t_{SRG}$, for different light intensity $I_{0}=0$ (**a**), $I_{0}=0.1$ (**b**) and $I_{0}=0.2$ (**c**). Reduced temperature T=0.2, trajectory of the CM of the chain (red), trajectory of the first (m=1) monomer (blue), and of the central (m=13) monomer (green).

The dynamics of a chain are characterized in Figure 3 by three trajectories corresponding to the motion of the CM of the chain (red), an edge monomer (m=1, blue), and a central one (m=13, green) in the full simulation period $t_{SRG}$. Three illumination setups are studied without illumination ($I_{0}=0$, panel (a)) and with illumination $I_{0}=0.1$ and $I_{0}=0.2$, panels (b) and (c), respectively. Qualitative analysis shows that light illumination has a strong impact on the dynamics. Namely, in its presence, all three types of trajectories penetrate a noticeably larger volume (with a linear size of over ten of the segment’s length) than for a dark (unilluminated) system. The trajectories of the CM and of the central monomer (m=13) penetrate similar volumes, in contrast to the edge monomers (m=1) which penetrate noticeablely larger volumes. This effect is the strongest in the system without illumination. The quantitative analysis is presented in the next sections.

#### 3.1.2. Case $I_{0}=0$

**Figure 4:**
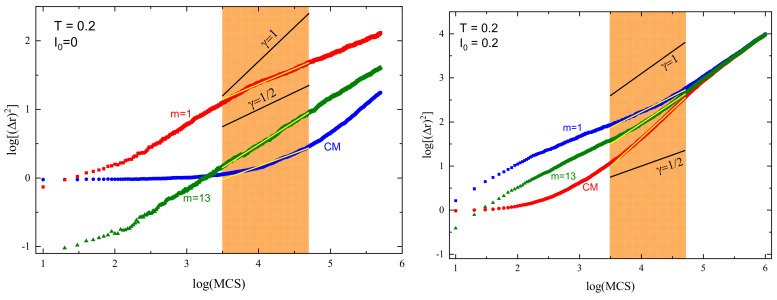
Log–log plot of averaged squared displacements ${\left(\Delta \vec{r}\right)}^{2}\left(t\right)$ without illumination (**left** panel) and with illumination $I_{0}=0.2$ (**right** panel) for the first (m=1, blue) and for the central (m=13, green) monomer and for CM (red). Reduced temperature T=0.2. Thin straight yellow lines show linear fits to the plots.

The azo-polymer chain without light illumination becomes a standard BFM chain. Exemplary log–log plots of displacements ${\left(\Delta r\right)}^{2}\left(t\right)$ of CM and selected monomers (m=1,13) are shown in Figure 4 (left panel). In the interval chosen for fitting (Section 2.3), marked orange in the plot, the system is not in the Rouse regime (this regime is still not present even for a much longer time, $10\;t_{SRG}$). We point out that the normal diffusion limit can be reached faster for a higher temperature (see Figure 6 in Ref. [8]).

**Figure 5:**
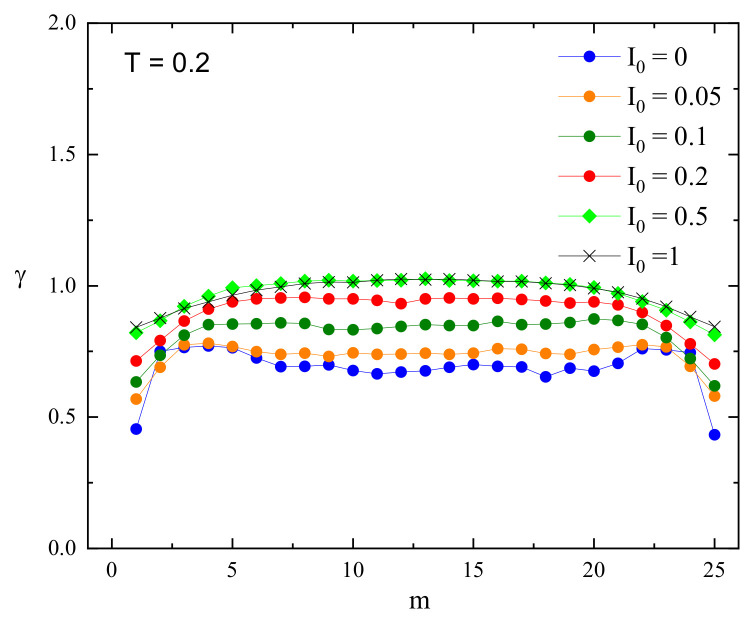
Exponents $\gamma_{m}$ calculated for single monomers for chosen intensities of homogeneous light illumination $I_{0}$. Reduced temperature T=0.2.

The dynamics of both monomers can be interpreted in terms of approximate power laws (Section 2.3). A rather surprising finding is that the slope for an edge monomer is slightly lower than its counterpart for a central monomer. This specific effect concerns only the edge monomer; see the blue curve in Figure 5. Namely, the exponent $\gamma_{1}$ for the edge monomer has a value slightly lower than 0.5, whereas the exponents for the remaining monomers are in the interval 0.67–0.75. One observes a weak maximum of $\gamma_{m}$ for a few monomers close to the edge. This effect was observed at infinite temperature [12]. The dynamics of the CM in the absence of light illumination cannot be interpreted in terms of a power law.

#### 3.1.3. Case $I_{0}>0$

**Figure 6:**
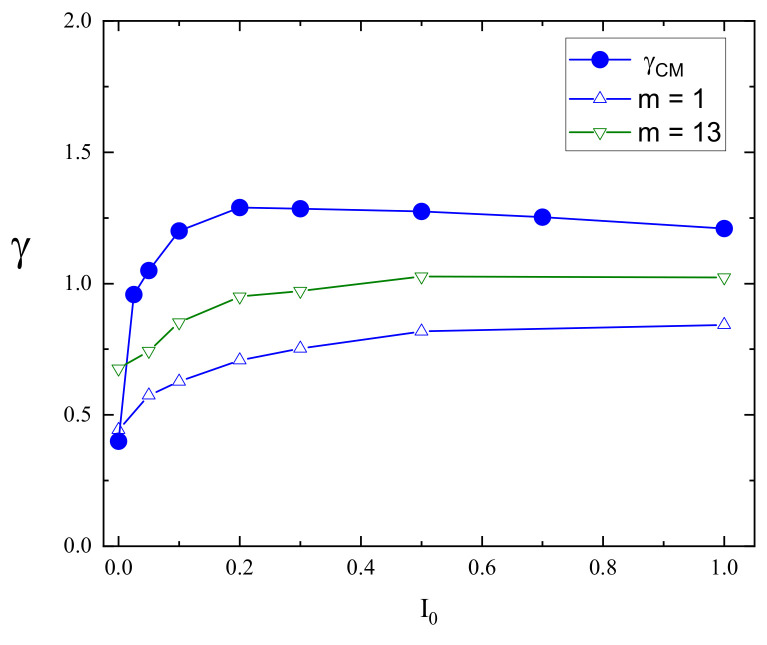
Exponents $\gamma_{CM}$, $\gamma_{1}$, and $\gamma_{13}$ in function of intensity of homogeneous light illumination $I_{0}$. Reduced temperature T=0.2.

The superdiffusive dynamics of CM of a single chain at constant illumination, reported in detail in Ref. [8], reflects the dynamics of the individual segments. Exemplary log–log plots of displacements ${\left(\Delta r\right)}^{2}\left(t\right)$ of CM and selected monomers (m=1,13) are shown in Figure 4 (right panel). The interval chosen for fitting the system is close to the Rouse regime, which sets in for times comparable to, but larger than $t_{SRG}$. The dynamics of both monomers and of CM can be tentatively characterized using power laws. Similarly, as in the case $I_{0}=0$, the slope for an edge monomer is slightly lower than its counterpart for a central monomer. The exponents $\gamma_{m}$ for a few chosen values of $I_{0}$ are shown in Figure 5. As the intensity $I_{0}$ increases, their values also increase. For strong illumination ($I_{0}=1$), all monomers, but few close to the edge, perform standard diffusion with $\gamma_{m}\approx 1$. The plots of $\gamma_{1}$ and $\gamma_{13}$ in function of $I_{0}$ are shown in Figure 6.

The dynamics of monomers are subdiffusive; nevertheless, a surprising effect is found for the dynamics of CM. Namely, the slope in Figure 4 (right panel) is larger than for the monomers and reads approximately $\gamma_{CM}\approx 1.25$ for $I_{0}=0.2$, indicating superdiffusion. Superdiffusive dynamics sets in practically for any non-zero intensity $I_{0}$; the subdiffusive regime is present only for a weak light illumination $I_{0}<0.04$.

**Figure 7:**
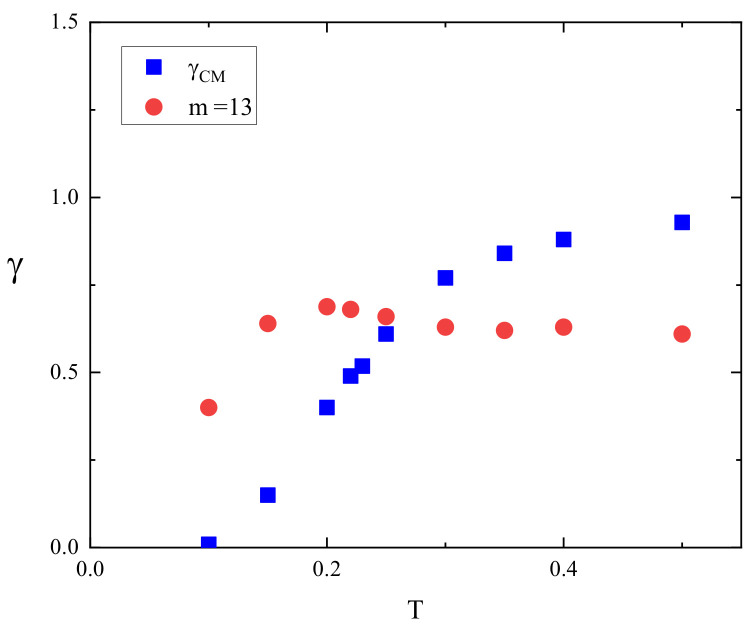
Exponents $\gamma_{CM}$ and $\gamma_{13}$ vs. reduced temperature for $I_{0}=0$.

Both qualitative and quantitative differences between the dynamics of CM and of the monomers can be inferred from the temperature dependence of the exponents $\gamma_{CM}$ and $\gamma_{13}$ in the absence of light illumination (Figure 7). The monotonic increase in $\gamma_{CM}$ was already reported [8]. For a sufficiently high temperature (above the glass transition temperature in the dense system), the dynamics of the CM of the chain approaches a normal diffusion in the time interval studied in this paper. The diffusion of the monomers is radically different. Exponent $\gamma_{13}$ weakly increases with the temperature and finally reaches a plateau $\gamma_{13}\approx 0.62$. The intersection of the plots of $\gamma_{CM}$ and $\gamma_{13}$ occurs for T≈0.26 for which $\gamma_{13}\approx \gamma_{CM}\approx 0.66$.

### 3.2. Inhomogeneous Illumination

#### 3.2.1. Trajectories

**Figure 8:**
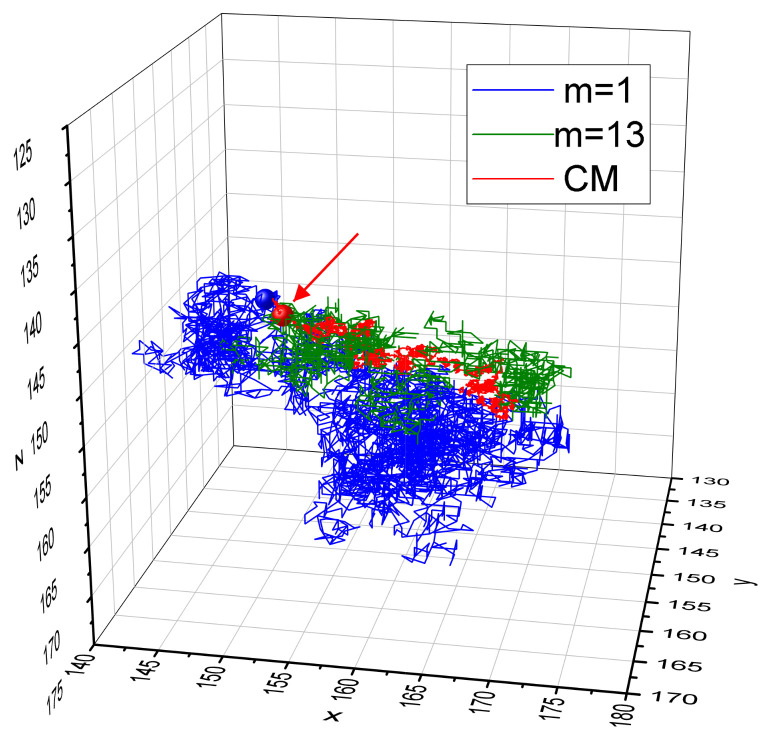
Exemplary trajectories of monomers m=1 (blue) and m=13 (green) and of the CM of the chain (red) in the full simulation period $t_{SRG}$, for $I_{0}=0.7$ and ∇I=0.005. Big red and blue dots denote the starting positions of CM (red arrow) and of monomer m=1, respectively. Reduced temperature T=0.25.

Exemplary trajectories of monomers m=1,m=13, and of the CM of the chain in the full simulation period $t_{SRG}$ are shown in Figure 8. Directed motion along the *x* axis is evident; initial positions of CM and of edge monomer are depicted by big spheres. As in the case of constant illumination, an edge monomer probes a much larger volume than the central one.

#### 3.2.2. Superdiffusive Dynamics

**Figure 9:**
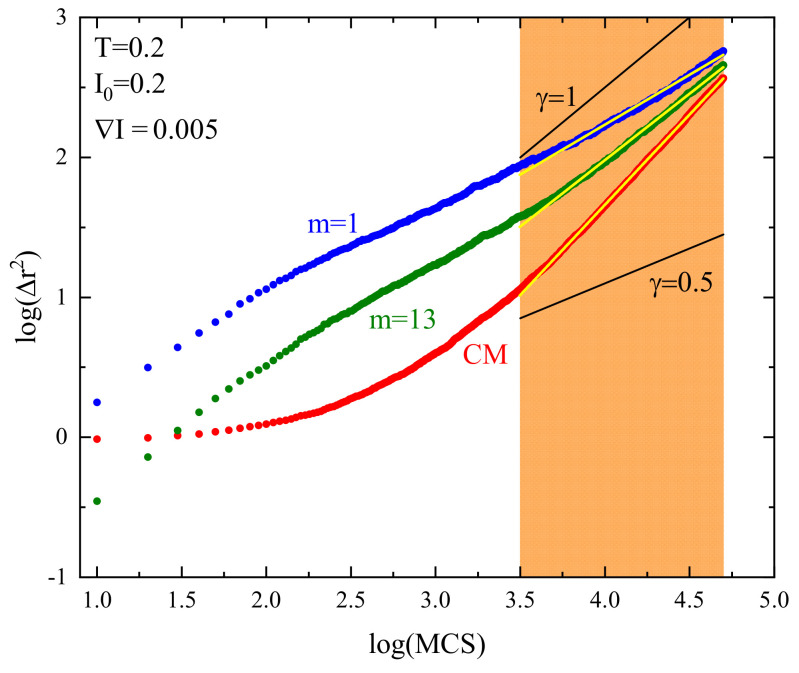
Log–log plot of averaged squared displacements ${\left(\Delta \vec{r}\right)}^{2}\left(t\right)$ for the first (m=1, blue), and for the central one (m=13, green) monomer and for CM (red). Reduced temperature T=0.2, ∇I=0.005, offset $I_{0}=0.2$. Thin straight yellow lines show linear fits to the plots.

**Figure 10:**
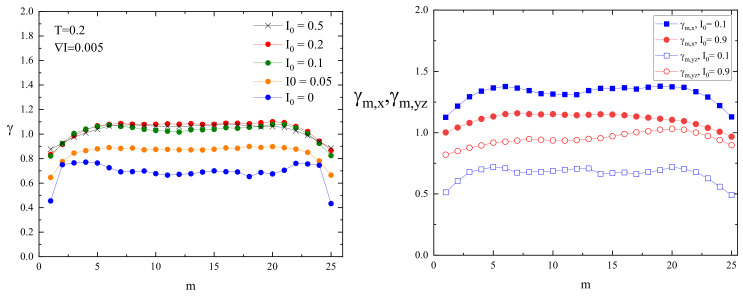
“Isotropic” exponents $\gamma_{m}$ (**left** panel) and “anisotropic” exponents $\gamma_{m,x},\gamma_{m,yz}$ (**right** panel) for chosen values of offset $I_{0}$. Reduced temperature T=0.2, ∇I=0.005.

Exemplary log–log plots of displacements ${\left(\Delta r\right)}^{2}\left(t\right)$ of CM and of selected monomers (m=1,13) for offset $I_{0}=0.2$ are shown in Figure 9. To avoid misunderstanding, we point out that displacement is characterized in three dimensions, not along a chosen axis. The dynamics of both monomers and of CM can be interpreted in terms of approximate power laws. Similarly, as in the cases studied before, the slope for an edge monomer is slightly lower than its counterpart for the central monomer. The exponents $\gamma_{m}$ for a few chosen values of offset $I_{0}$ are shown in Figure 10. As in previous cases, the exponents for few monomers close to the edge are smaller than those for central monomers. The exponents increase with increasing offset.

The plots of exponents $\gamma_{m}$ for constant illumination (Figure 5) and for an inhomogeneous illumination (Figure 10, left panel) are qualitatively similar but, on a quantitative level, display an important difference. Namely, in the former case, the dynamics of monomers are subdiffusive and, for strong illumination, diffusive. On the contrary, in the presence of a gradient, the increase in offset is accompanied by a crossover of dynamical regimes: from subdiffusive, through diffusive, to weak superdiffusive. Moreover, for sufficiently high values of the offset $I_{0}$ subdiffusive and superdiffusive dynamics are present in various parts of the chain.

The characterization of the dynamics of the monomers presented above conceals the strongly non-isotropic character of the diffusion. In the remaining part of this section we study the diffusion along three directions separately.

The dynamics along the direction of the gradient is characterized in terms of exponents $\gamma_{CM,x}$ (for CM)) and $\gamma_{m,x}$ (for monomers), and in perpendicular directions (y,z)-by averaged over directions y,z exponents $\gamma_{CM,yz}=\left(\gamma_{CM,y}+\gamma_{CM,z}\right)/2$ and $\gamma_{m,yz}=\left(\gamma_{m,y}+\gamma_{m,z}\right)/2$. The plots of exponents for the monomers are shown in Figure 10, right panel. All the monomers display the superdiffusion along the direction of the gradient (less pronounced for edge monomers); the exponents decrease with increasing offset $I_{0}$. On the contrary, the dynamics of the monomers along directions perpendicular to the gradient are subdiffusive, approaching normal diffusion as $I_{0}$ increases.

**Figure 11:**
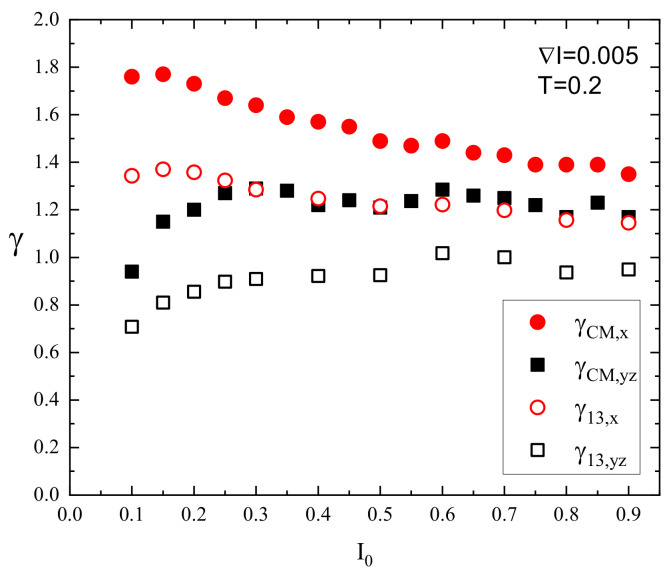
Exponents $\gamma_{CM,x}$ (red circles), $\gamma_{CM,yz}$ (black squares), $\gamma_{13,x}$ (empty circles), $\gamma_{13,yz}$ (empty squares) calculated for different intensity $I_{0}$ with ∇I=0.005. Reduced temperature T=0.2.

The impact of an offset intensity $I_{0}$ on “isotropic” and “anisotropic” exponents for CM and central monomer (m=13) is shown in Figure 11. The center of mass displays a superdiffusion which, at low values of an offset, is close to ballistic. The exponent $\gamma_{CM,x}$ decreases linearly as $I_{0}$ increases; this behavior was reported in Ref. [8]. The dynamics of monomers are qualitatively similar; they are superdiffusive, the exponent $\gamma_{13,x}$ has smaller values than its counterpart for CM, and it also decreases linearly with increasing offset. The dynamics in directions y,z are both qualitatively and quantitatively different. Firstly, the exponents $\gamma_{CM,yz}$ and $\gamma_{13,yz}$ increase with $I_{0}$. Secondly, whereas the CM undergoes superdiffusive dynamics (Ref. [8]), the monomers perform subdiffusive motion; the plots of exponents are (approximately) shifted by $\Delta \gamma_{yz}\approx 0.30$.

## 4. Discussion

In this paper, the generalized Bond Fluctuation Model, destined for modeling nonlinear optical light-driven phenomena in host–guest systems, is used to characterize the complex dynamics of monomers in chains functionalized with azo-dyes, in the presence of spatially inhomogeneous light illumination. The paper presents a number of facts related to the subdiffusive, diffusive, and superdiffusive dynamics of monomers rather than an attempt to provide theoretical interpretations. The results shed some light on the dynamics of model polymer chains on temporal scales typical for light-driven nonlinear optical phenomena, such as Surface Relief Gratings build-up [7] or all-optical poling [27]. The rather technical discussion of the results accompanies their presentation in the main text. Here, we discuss them in a wider context.

There are three main results of this study. The first one refers to characteristic temporal scales. We found that the light-driven dynamics of monomers are complex (sub- or superdiffusive) in the time interval typical for the build-up of Surface Relief Grating. Correspondingly, this effect cannot be modeled via the long-time limits of Rouse-like models.

Next, we identified the parameters responsible for the crossover between dynamical regimes for monomers in a single chain. A crossover from subdiffusion to normal diffusion is driven by light intensity in the case of constant illumination (Figure 5) and by offset intensity in the case of perpendicular motion in the presence of an inhomogeneous light illumination (Figure 11). Of special interest is the crossover from subdiffusion through normal diffusion to superdiffusion, driven by offset $I_{0}$ in the presence of an inhomogeneous light illumination (Figure 10, left panel). Namely, the studies of the dynamics of the center of mass of the chains [8] led to the conclusion that the directed mass transport results from the coexistence of subdiffusion, normal diffusion, and superdiffusion in various parts of the polymeric system. In this study, we showed (Figure 10, left panel) that those dynamical regimes can coexist in a single chain as well.

Finally, we observed a noticeable dispersion of the values of the exponents. Most of the monomers (the “central” ones) have close values to exponent γ. On the contrary, the remaining (“edge”) monomers have lower values of γ (Figure 5, Figure 6 and Figure 10). This result is rather counterintuitive because the edge monomers probe a larger volume in the space than the central ones (Figure 3 and Figure 8). We are of the opinion that the number of edge monomers does not scale with the length of the chain and, in consequence, longer polymer chains display very similar dynamics for practically all monomers. This study is in progress now.

The theoretical modeling of the light-driven motion of azo-polymer chains constitutes a challenge. Complex dynamics can be modeled in the framework of generalized central limit theorems for independent identically distributed (i.i.d.) random variables displaying scaling laws. We found that the dynamics of azo-polymer chains exposed to light illumination in the time interval of interest for physical applications cannot be modeled in this way. Consider the case of strong constant illumination $I_{0}=1$, Figure 5. Most of the monomers perform normal (Gaussian) diffusion (γ=1). If few edge monomers are neglected, the central limit theorem predicts also a normal diffusion for the center of mass, on the condition that random variables which characterize the motion of the monomers are i.i.d. random variables. This is not the case for the chain, because the center of mass performs a superdiffusion with γ≈1.25 for $I_{0}=1$ (Figure 6). Let us point out that for longer time intervals, when the normal diffusion sets in, this approach becomes valid. For example, in the case of constant light illumination $I_{0}=0.2$, Figure 4 (right panel) this regime sets in for times slightly larger than $t_{SRG}$.

Finally, as a side effect, this study offers an approximate localization of glass transition temperature $T_{g}$. Namely, the plots of exponents $\gamma_{CM}$ and $\gamma_{13}$ (in the absence of light illumination, Figure 7) intersect in the middle of the interval 0.225<T<0.3 in which the glass the transition temperature was localized [27]. This observation is rather surprising, because the glass transition is a cooperative process in a bulk.

## 5. Conclusions

This study deepens the actual knowledge [8] related to the anomalous diffusion of a model azo-polymer chain interacting with a spatially inhomogeneous laser light by providing quantitative characteristics (approximate scaling laws) of complexity for the monomers in a single chain.

The relation between the temporal scales characterizing the time span for physical processes and, on the other hand, the time when the standard diffusion of polymer chains sets in, is of primary importance for the modeling. It seems that the former is shorter, leading to overall complex dynamics.

The first results indicate that the relation between the anomalous diffusion of the center of mass of a chain and of anomalous diffusion of the monomers is complicated and cannot be directly analyzed using generalized central limit theorems. Potentially, other mathematical tools of complex systems can find applications here. On the other hand, simplified approaches can be successfully used, such as the Continuous Time Random Walk [8]. We are of the opinion that a generalization of this model to three dimensions, with restrictions due to allowed distances between the monomers, might offer a reasonable starting point for modeling the dynamics of the system of azo-chains. This study is in progress now.

## Data Availability

Not applicable.
